# Viral Load Difference between Symptomatic and Asymptomatic COVID-19 Patients: Systematic Review and Meta-Analysis

**DOI:** 10.3390/idr13030061

**Published:** 2021-07-16

**Authors:** Marco Zuin, Valentina Gentili, Carlo Cervellati, Roberta Rizzo, Giovanni Zuliani

**Affiliations:** 1 Department of Translational Medicine, University of Ferrara, 44121 Ferrara, Italy; marco.zuin@edu.unife.it (M.Z.); crvcrl@unife.it (C.C.); zlngnn@unife.it (G.Z.); 2 Department of Chemical, Pharmaceutical and Agricultural Sciences, University of Ferrara, 44121 Ferrara, Italy; roberta.rizzo@unife.it

**Keywords:** COVID-19, viral load, real-time RT-PCR, SARS-CoV-2

## Abstract

We conducted a systematic review and meta-analysis to investigate the possible difference in the SARS-CoV-2 viral load between asymptomatic and symptomatic COVID-19 patients. Preferred Reporting Items for Systematic Reviews and Meta-Analyses guidelines were followed in abstracting data and assessing validity. We searched MEDLINE, Scopus, Web of Science and Google Scholar for all investigations in the English language, reporting data on the threshold cycle (C_t_) from real-time RT-PCR assays for the RNA-dependent RNA polymerase (*RdRp*), envelope (*E*) and nucleocapsid (*N*) SARS-CoV-2 genes in asymptomatic and symptomatic COVID-19 patients. Results: Overall, 703 COVID-19 patients (553 symptomatic and 150 asymptomatic) were analyzed. Five investigations reported the mean age of patients, evidencing that asymptomatic patients were younger than symptomatic patients (34.0 vs. 40.3 years, respectively). Pooled data regarding the levels of expression of the *RdRp* gene revealed no significant difference between symptomatic and asymptomatic subjects. Similarly, no differences were observed comparing the mean C_t_ values for the *E* and *N* genes. Based on real-time RT-PCR data, no differences exist in the viral load between symptomatic and asymptomatic COVID-19 subjects considering C_t_ values for *RdRp*, *E* and *N* genes’ expression. Asymptomatic subjects may represent a reservoir of the infection and significantly contribute to the maintenance of the pandemic.

## 1. Introduction

Since the beginning of the COVID-19 pandemic, SARS-CoV-2 reverse transcription polymerase chain reaction (RT-PCR) testing has played a pivotal role in both diagnosis and epidemiological analyses worldwide [1]. In clinical practice, RT-PCR assays generally target the RNA-dependent RNA polymerase (*RdRp*), envelope protein (*E*) or nucleocapsid protein (*N*) genes [2]. Notably, as it has been demonstrated since the early stage of the outbreak, the spread of SARS-CoV-2 infection has been largely maintained by asymptomatic individuals [3,4,5]. Over the latest months, some investigations analyzing the dynamic of COVID-19 transmission have reported no differences in viral loads between asymptomatic and symptomatic patients, determining substantial containment challenges [5]. However, a direct demonstration of this aspect in COVID-19 patients, until now, has only been reported in the form of isolated reports and never analyzed comprehensively.

The aim of the present study was to determine the existence, if any, of different viral loads between symptomatic and asymptomatic COVID-19 patients, conducting a meta-analysis based on the available data published in the literature.

## 2. Methods

### 2.1. Search Strategy

This study followed the Preferred Reporting Items for Systematic Reviews and Meta-analyses (PRISMA) reporting guidelines (Supplementary File S1) [6]. Data were obtained searching PubMed-MEDLINE and Scopus for all investigations from inception to 1 March 2021, reporting data regarding the threshold cycle (C_t_) from RT-PCR assays for RNA-dependent RNA polymerase (*RdRp*), envelope protein (*E*) or nucleocapsid protein (*N*) genes in asymptomatic and symptomatic COVID-19 patients.

### 2.2. Outcomes

The outcome of this study was to assess potential differences in the C_t_ values of *RdRp*, *E* and *N* genes, among symptomatic and asymptomatic COVID-19 patients. These molecular targets were selected according to the current WHO recommendations and since they have been the most investigated and reported parameters regarding COVID-19 infection [2,7,8].

### 2.3. Study Selection

The selection of studies to be included in our analysis was independently conducted by 2 authors (M.Z. and C.C.) in a blinded fashion. Any discrepancy in study selection was resolved consulting a third author (G.Z.). The following MeSH terms were used for the search: “COVID-19” OR “SARS-CoV-2” AND “Viral load” AND “Asymptomatic”. Moreover, we searched the bibliographies of target studies for additional references. Case reports, review articles, abstracts, editorials/letters and case series with less than 10 participants were excluded. Data extraction was independently conducted by 2 authors (M.Z. and G.Z.). Studies were included in the present analysis if: (1) they provided a comparison for the C_t_ values of *RdRp, E* and *N* genes between symptomatic and asymptomatic patients; (2) data were presented as mean and standard deviation; if not provided in this form, or the dataset was not available in the Supplementary Material or online data repository, we asked for the missing information by contacting the original study investigators. Data provided as mean and related interquartile range (IQR) were excluded since we were not able to assess if they had a normal distribution and therefore calculate the relative standard deviation. Articles not in the English language or without a full text available were excluded. Asymptomatic patients were defined as those that did not have symptoms at the time of swab testing and/or did not develop symptoms afterwards.

### 2.4. Data Extraction

For each investigation included in the final analysis, the following items were extracted: first author, sample size (number of symptomatic and asymptomatic patients), mean age of each group, type of sample, RNA extraction and assay used. The quality of included studies was graded using the Newcastle–Ottawa quality assessment scale [9].

### 2.5. Statistical Analysis

Continuous variables were expressed as mean and standard deviation (SD) or only as a mean if SDs were not provided in the original study. Data were analyzed using the Mantel–Haenszel method, and the standardized mean difference (SMD) and relative 95% confidence intervals (CI) were calculated. Statistical heterogeneity between groups was measured using the Higgins I^2^ statistic. Specifically, I^2^ = 0 indicated no heterogeneity, while we considered low, moderate and high degrees of heterogeneity based on the values of I^2^ of <25%, 25–75% and above 75%, respectively. Significant heterogeneity was assumed when the *p*-value was <0.1 or the I^2^ value was >50%. A random-effect model (DerSimonian–Laird) was used if the heterogeneity test was statistically significant or I^2^ > 50%; otherwise, a fixed-effect model was carried out. To evaluate potential bias, both Egger’s test and funnel plots were computed. To further appraise the impact of potential baseline confounders, a meta-regression analysis was conducted using the patients’ demographic characteristics (i.e., age and gender) as moderator variables. A *p*-value < 0.05 was considered statistically significant. All meta-analyses were conducted using Comprehensive Meta-Analysis software, version 3 (Biostat, Englewood, NJ, USA).

## 3. Results

### 3.1. Search Results and Study Characteristics

**Figure 1:**
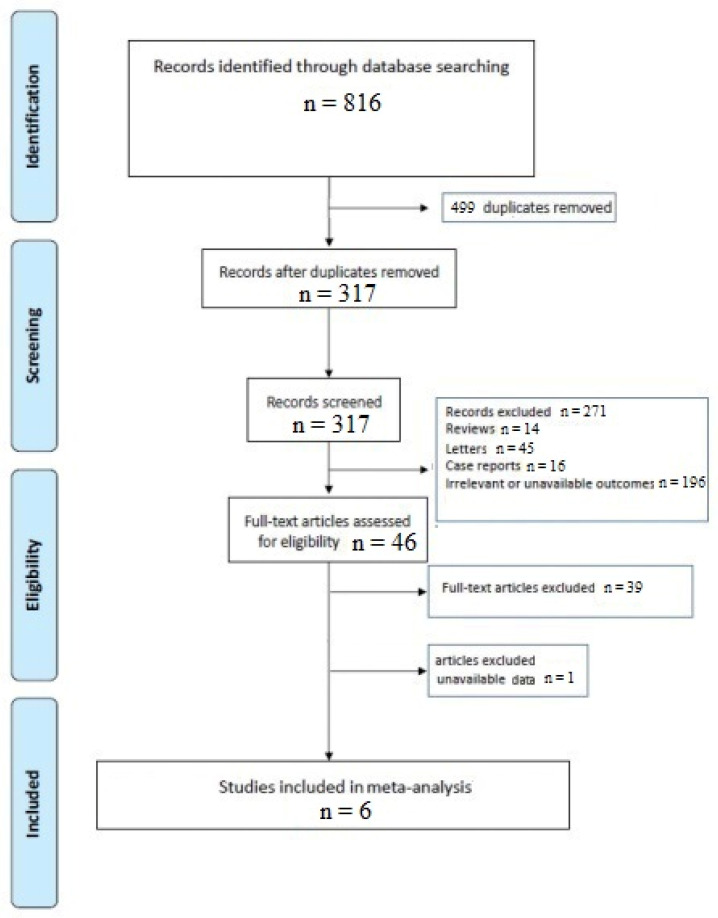
PRISMA flow chart.

A total of 317 articles were retrieved after excluding duplicates. The initial screening excluded 271 articles because they did not meet the inclusion criteria, leaving 46 studies to assess for eligibility. After evaluation of the full-text articles, 39 were excluded for not meeting the inclusion criteria, and one article was excluded since we were not able to obtain the missing data, leaving 6 investigations suitable for the analysis (Figure 1) [5,10,11,12,13,14].

The baseline characteristics of the included studies are shown in Table 1. Overall, 703 COVID-19 patients (553 symptomatic and 150 asymptomatic subjects) were analyzed. Five investigations reported the mean age of patients, evidencing that asymptomatic patients were younger than symptomatic patients (34.0 vs. 40.3 years, respectively). Unfortunately, no data regarding the reason why asymptomatic patients were tested were reported.

All the revised investigations reported the mean C_t_ values for *RdRp* in both symptomatic and asymptomatic COVID-19 patients, whereas five and four investigations also presented data regarding the mean C_t_ values for the *E* and *N* genes, respectively. According to the NOS, the studies included in the meta-analysis resulted in being of a moderate-high quality.

### 3.2. Differences in RdRp between Asymptomatic and Symptomatic COVID-19 Patients

**Figure 2:**
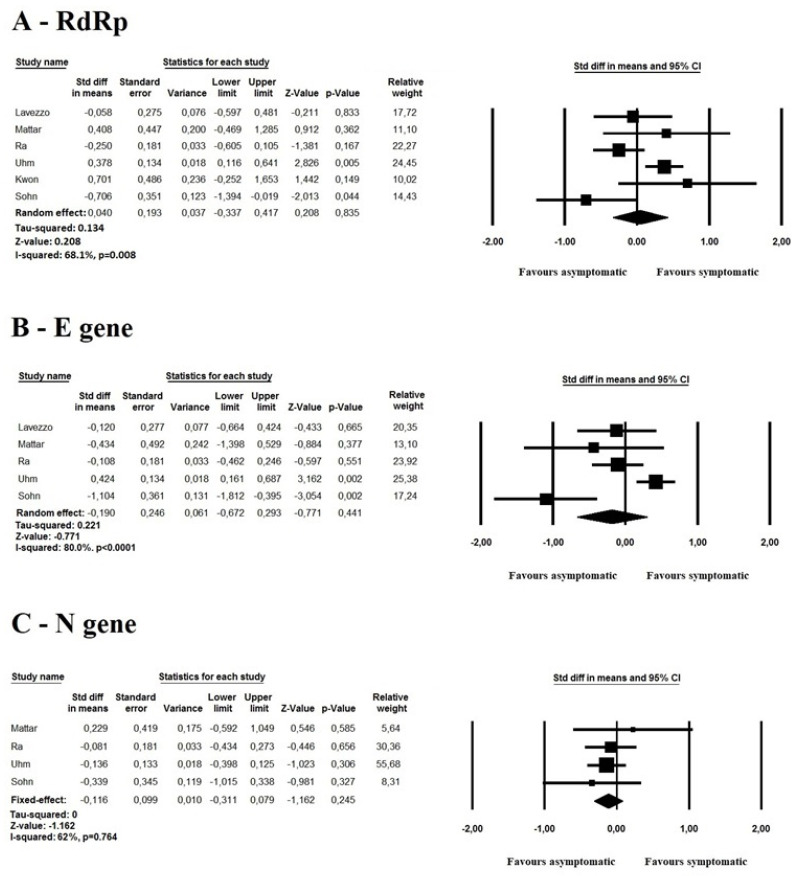
Forest plots of the standardized mean difference (SMD) between asymptomatic and symptomatic SARS-CoV-2 patients for the *RdRp* (Panel A), *E* (Panel B) and *N* genes (Panel C). The standard mean difference (Std diff in means), relative standard error and the corresponding 95% confidence interval are reported for each study. The relative weight of the studies in each model is also reported.

Pooled data of the *RdRp* gene [5,10,11,12,13,14] revealed, using a random-effect model, no significant differences in the mean C_t_ value for *RdRp* between symptomatic and asymptomatic subjects (SMD 0.040, 95% CI −0.037 to 0.417 *p* = 0.83, I^2^: 68.1%) (Figure 2, Panel A).

### 3.3. Differences in the E Gene between Asymptomatic and Symptomatic COVID-19 Patients

Considering the five investigations reporting the mean C_t_ values for the *E* gene [5,10,12,13,14], which were based on 714 patients (161 asymptomatic and 553 symptomatic), the random-effect model showed no significant differences (SMD −0.190, 95% CI −0.672 to 0.293 *p* = 0.441, I^2^: 80%) among the two groups (Figure 2, Panel B).

### 3.4. Differences in the N Gene between Asymptomatic and Symptomatic COVID-19 Patients

Four studies (661 patients, 135 asymptomatic and 526 symptomatic) [10,12,13,14] compared the mean C_t_ values of the *N* gene among asymptomatic and symptomatic SARS-CoV-2 patients. Again, no differences were noted between the two groups (SMD: −0.116, 95% CI: −0.311 to 0.079, *p* = 0.24, I^2^: 62.0%) (Figure 2, Panel C).

### 3.5. Meta-Regression

Meta-regression analysis evidenced that the differences in *RdRP*, *E* and *N* genes between asymptomatic and symptomatic patients were influenced by age (*p* = 0.03, *p* = 0.01 and *p* = 0.04 for *RdRP*, *E* and *N* genes, respectively) and female gender (*p* = 0.01, *p* = 0.002 and *p* = 0.01, for *RdRP*, *E* and *N* genes, respectively).

### 3.6. Assessment of Publication Bias

**Figure 3:**
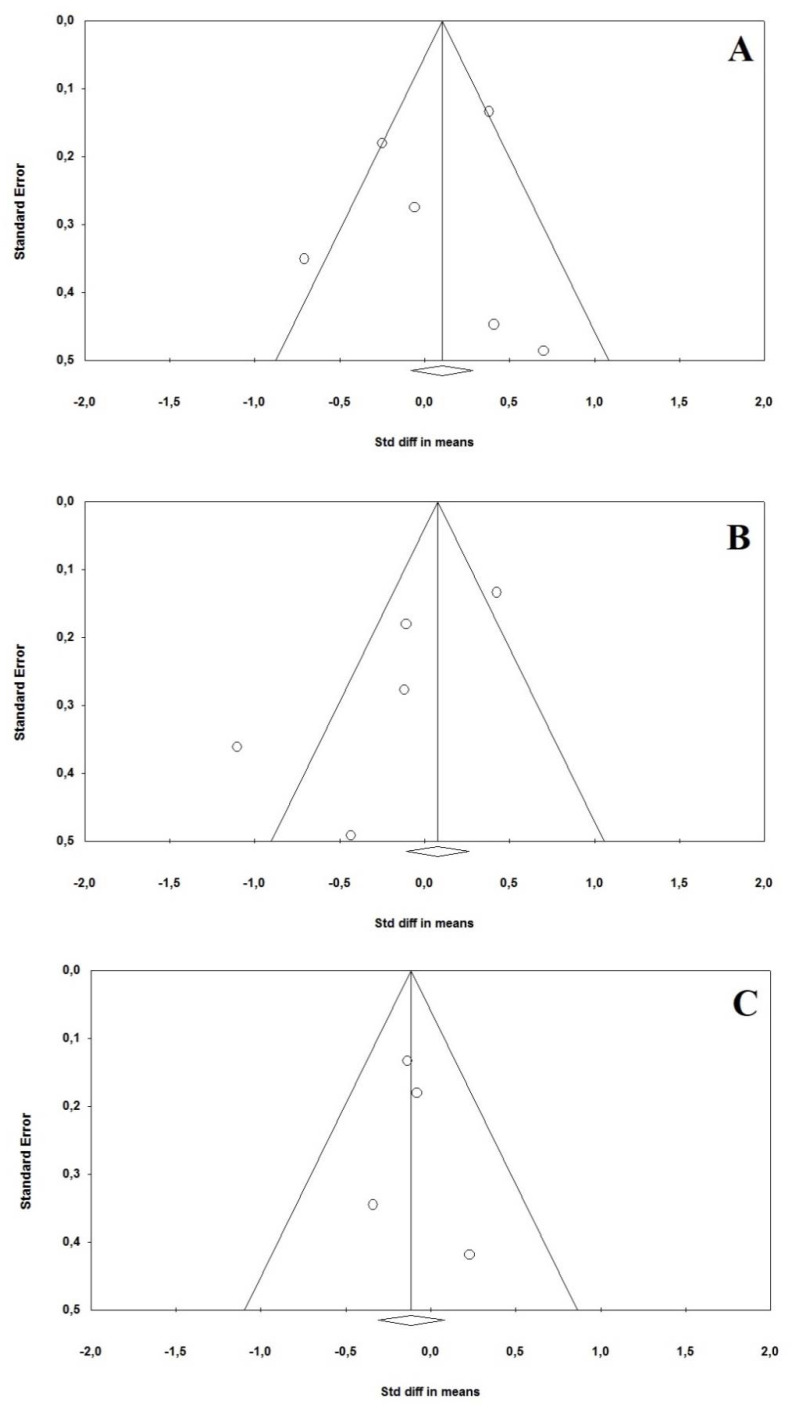
Funnel plots for the standardized mean difference (SMD) between asymptomatic and symptomatic SARS-CoV-2 patients for the *RdRp* (Panel **A**), *E* (Panel **B**) and *N* genes (Panel **C**). Each study is represented by a circle. Diagonal lines represent the 95% confidence limits around the summary treatment effect, i.e., [summary effect estimate − (1.96 × standard error)] and [summary effect estimate + (1.96 × standard error)], for each standard error on the vertical axis. These show the expected distribution of studies in the absence of heterogeneity or of selection biases: in the absence of heterogeneity, 95% of the studies should lie within the funnel defined by these straight lines. As these lines are not strict 95% limits, they are referred to as “pseudo-95% confidence limits”. The rhomboid and relative vertical line represent the summary estimate of the treatment effect, derived using a random-effect meta-analysis model.

Egger’s tests revealed no evidence of publication bias in estimating the SMD for *RdRp* (t = 0.411, *p* = 0.702), *E* (t = 2.498, *p* = 0.085) and *N* (t = 0.353, *p* = 0.752). A visual assessment using the funnel plots for the same outcomes is presented in Figure 3 (Panels A, B and C for *RdRp*, *E* and *N* genes, respectively).

## 4. Discussion

Our analysis evidenced that symptomatic and asymptomatic SARS-CoV-2 patients have the same viral load since no differences were observed in terms of mean C_t_ values for the *RdRp, E* and *N* genes among the two groups. As it was recommended by the World Health Organization (WHO), the *RdRp*, *E* and *N* genes represent the targets for the detection of SARS-CoV-2, specifically the *E* gene for first-line screening, the *RdRp* gene for the confirmatory assay and the *N* gene for the additional confirmatory assay [15].

*RdRp*, also known as nsp12, has a fundamental role in both the replication and transcription of SARS-CoV-2, using the cofactors nsp7 and nsp8 [16,17]. The envelope (E) protein is the smallest of all the structural proteins and is involved in both the assembly and release of the virions [18], also acting as a viroporin [19]. However, the quantification of the *E* gene, per se, results in being more sensitive compared to *RdRp* [2]. Indeed, some investigators have observed that using the *E* gene as a target in addition to *RdRp* significantly increases the sensitivity of viral identification by approximately 12% [1]. However, assessing whether the *E* gene should be evaluated alone or with *RdRp*, as well as the diagnostic accuracy of the different approaches to identifying SARS-CoV-2, is beyond the aim of our manuscript. The nucleocapsid protein (N), which is produced at high levels in infected cells, plays an important role in the packaging of viral RNA into ribonucleocapsid and enhances the efficiency of viral RNA transcription, through the interaction with the M protein and viral genome [19,20].

Doubtless, the prompt and diffuse identification of asymptomatic patients represents a major concern in the containment of the infection worldwide, considering that these subjects are generally identified incidentally or as a part of contact tracing performed to reconstruct the transmission chains.

The high heterogeneity observed was influenced by age and gender, as evidenced by the meta-regression. These findings are in accordance with some previous analyses demonstrating that the viral load increases with age [21] and is higher in females [22]. However, we also cannot use underlying comorbidities, such as arterial hypertension and diabetes, as moderator variables, which have been related to a slower viral shedding [23,24]. Moreover, the intrinsic and unmodifiable heterogeneities existing among laboratories have also doubtlessly influenced our results. In the same manner, minor differences in the timing of performing the nasopharyngeal swab, and the different stratifications of patients proposed in the articles reviewed may have distorted the viral load assessment. It should be noted that our analysis was not able to assess the different viral variants among the population analyzed, since these data were not provided by original studies as viral genomic sequencing was not yet performed. In this regard, further analyses must be urgently performed.

As a matter of fact, very few investigations have compared the viral load between asymptomatic and symptomatic COVID-19 patients. The interest in this topic resulted in being relevant only during the first phase of the outbreak. Therefore, the pivotal role of often undetected asymptomatic subjects in the spread of infection, which remains underestimated in daily epidemiological reports, has direct implications in the spread of infection [25].

Our results reinforce some important concepts which have a direct implication in the containment of the disease. First, asymptomatic patients are generally younger compared to those who are symptomatic. Second, these subjects represent a reservoir of the infection and significantly contribute to the maintenance of the pandemic. Third, due the similar viral load, asymptomatic patients must be managed as symptomatic ones, with adequate restrictive measures and isolation to avoid the perpetuation of the infection. Despite the previous analyses which reported a faster viral clearance in asymptomatic compared to symptomatic patients, it is important to underline that our analysis considers the C_t_ values during the first days of infection in all the studies reviewed. Furthermore, it is important to keep in mind that rebounds of C_t_ values in asymptomatic patients have been described not as an uncommon event [14,26].

### Limitations

Our analysis has several limitations. Firstly, only a few investigations have analyzed and compared the C_t_ values for the *RdRp*, *E* and *N* genes in COVID-19 patients, thus limiting the number of studies included in the analysis. Secondly, the high heterogeneity observed, which probably depends on the participants’ inclusion criteria, design of the studies reviewed and the unmodifiable intra-assay, inter-assay, intra-laboratory and inter-laboratory heterogeneities, including the RNA extraction method and the probe-based technology used, may have limited the Ct value comparison and could have led to weak conclusions. Finally, an investigation was excluded from the meta-analysis since the data were presented as means and the relative interquartile ranges, and we were not able to obtain the results in the required form to perform the meta-analysis. However, also in this case, no difference was observed in the mean C_t_ values of *RdRp* between asymptomatic and symptomatic subjects [27].

## 5. Conclusions

Based on RT-PCR data, no differences exist in the viral load between symptomatic and asymptomatic COVID-19 subjects considering the C_t_ values for the *RdRp*, *E* and *N* SARS-CoV-2 genes. Our finding of a comparable viral load in the two populations analyzed supports the evidence that SARS-CoV-2 has a potentially high person-to-person transmission rate, even for asymptomatic subjects. For this reason, compliance with the technical guidelines for COVID-19 is fundamental to contain the viral spread until prophylactic or therapeutic treatments are available worldwide.

## Figures and Tables

**Table 1:** General characteristics of the studies reviewed.

Author	Country	N° of Asymptomatic (Mean Age)	N° of Symptomatic (Mean Age)	Setting	Sample	RNA Extraction	Assay **	C_t_ Value RdRp	C_t_ Value E Gene	C_t_ Value N Gene	NOS
Lavezzo et al. [5] °°	Italy	26 [NR]	27 [NR]	C	Oropharyngeal and nasopharyngeal	Magna Pure 96 System (Roche Diagnostic)	One Step Real Time kit (Thermo Fisher Scientific)	X	X	-	8
Kwon et al. [11]	Republic of Korea	6 [32.8 ± 9.5]	17 [45.8 ± 17.0] *	H	Nasopharyngeal	Magna Pure 96 System (Roche Diagnostic)	Allplex 2019-cCOVB Assay Kit (Seegene)	X	-	-	7
Ra et al. [12]	Republic of Korea	39 [25.0]	144 [36.5]	C	Oropharyngeal and nasopharyngeal	Magna Pure 96 System (Roche Diagnostic)	Allplex 2019-cCOVB Assay Kit (Seegene)	X	X	X	8
Uhm et al. [13]	Republic of Korea	68 [33.5]	328 [44.0]	H	Nasopharyngeal	NX-48 (Genolutio)	PowerCheck 2019-nCOv assay (Kogene Biotech)	X	X	X	8
Mattar et al. [10]	Colombia	17 [29.8]	17 [44.5]	C	Nasopharyngeal	Column-based (NR)	AllplexTM, 2019-nCoV assay, Korea	X	X	X	8
Sohn et al. [14]	Republic of Korea	11 [35.2 ± 17.6]	37 [31.8 ± 13.7]	C	Nasopharyngeal and salivary	QIAGEN kit (Qiagen)	AllplexTM, 2019-nCoV assay, Korea	X	X	X	8

* Defined as moderate severity. ** All assays are probe-based. C_t_: cycle threshold; NOS: Newcastle–Ottawa quality assessment scale; NR: not reported; C: community patients; H: hospitalized patients. °° Obtained from the online data repository of the study.

## Data Availability

Not applicable.

## Supplementary Materials

The following are available online at https://www.mdpi.com/article/10.3390/idr13030061/s1, File S1: PRISMA checklist.
